# Prevalence and Abundance of Florfenicol and Linezolid Resistance Genes in Soils Adjacent to Swine Feedlots

**DOI:** 10.1038/srep32192

**Published:** 2016-08-30

**Authors:** Qin Zhao, Yang Wang, Shaolin Wang, Zheng Wang, Xiang-dang Du, Haiyang Jiang, Xi Xia, Zhangqi Shen, Shuangyang Ding, Congming Wu, Bingrui Zhou, Yongning Wu, Jianzhong Shen

**Affiliations:** 1 Beijing Advanced Innovation Center for Food Nutrition and Human Health, College of Veterinary Medicine, China Agricultural University, Beijing 100193, China; 2 College of Animal Husbandry and Veterinary Medicine, Henan Agricultural University, Zhengzhou 450002, China; 3 Department of Veterinary Microbiology and Preventive Medicine, College of Veterinary Medicine, Iowa State University, Ames, IA 50011, USA; 4 Shanxi Key Laboratory of Ecological Animal Science and Environmental Medicine, Shanxi Agricultural University, Taigu 030801, China; 5 The Key Laboratory of Food Safety Risk Assessment, Ministry of Health and China National Center for Food Safety Risk Assessment, Beijing 100021, China

## Abstract

Florfenicol is extensively used in livestock to prevent or cure bacterial infections. However, it is not known whether the administration of florfenicol has resulted in the emergence and dissemination of florfenicol resistance genes (FRGs, including *fexA*, *fexB*, *cfr*, *optrA*, *floR*, and *pexA*) in microbial populations in surrounding farm environments. Here we collected soil samples for the detection of FRGs and the residue of florfenicol from six swine farms with the record of florfenicol usage. Quantitative polymerase chain reaction and metagenomic sequencing revealed a significantly higher relative abundance of FRGs in the soils adjacent to the three swine farms where florfenicol was heavily used compared with the other sites. Meanwhile, the detectable levels of florfenicol were also identified in soils from two of these three farms using ultra-performance liquid chromatography tandem mass spectrometry. It appears that amount of florfenicol used on swine farms and the spreading of soils with swine waste could promote the prevalence and abundance of FRGs, including the linezolid resistance genes *cfr* and *optrA*, in adjacent soils, and agricultural application of swine manure with florfenicol may have caused a residual level of florfenicol in the soils.

The growing number of bacterial strains carrying antibiotic-resistance genes (ARGs) poses a significant risk to both animal and human health. It is widely believed that the increased abundance of ARGs in the environment is contributing to the emergence of multidrug-resistant pathogens, which might lead to the failure of antibiotic treatment of bacterial infections^1^. In China, approximately 210,000 metric tons of antibiotics are produced per year, of which, 97,000 metric tons are used for therapy and growth promotion in animal husbandry^2^. It is estimated that approximately more than half of antibiotics are not absorbed in the animal gut, and subsequent selection has given rise to increasing numbers of resistant bacteria in the gastrointestinal tract, providing a potential reservoir for antibiotic resistance genes^3^. Furthermore, it is likely that many of the unabsorbed antibiotics, along with bacterial antibiotic resistance genes, are excreted into the environment via feces from livestock animals^3^. These antibiotics and their associated antibiotic resistance genes may accumulate in soils after repeated application of manure^3^,^4^. The residue of antibiotics even at low concentrations in the environment is likely to impose selective pressures on environmental microorganisms, which might induce the emergence of diverse ARGs and promote the evolution of novel genes conferring certain antibiotic resistance mechanisms^4^,^5^,^6^,^7^,^8^.

Soil is the predominant reservoir for bacteria harboring genes associated with the antibiotic resistance, with a number of antibiotic resistance determinants identified from soil bacteria^9^ and various resistance bacteria were cultured from soil samples^10^. Growing evidence shows that antibiotics, along with considerable numbers of antibiotic-resistant bacteria, ARGs, and associated mobile genetic elements, are being disseminated into agricultural soils through frequent manure waste application and contamination^11^,^12^,^13^. Fang *et al*. reported that the contamination of antibiotics and ARGs were detected in soils and the samples were collected from the actual field which had been treated with chicken manure for long term^6^. Moreover, the abundance of antibiotics and ARGs increased with the extension of greenhouse planting years^6^. This is likely to result in the ubiquitous pollution of antibiotic resistance in agricultural soils, while posing significant potential risks to the environment and public health. In addition, an ever-increasing number of novel functional resistance genes are being identified from environmental samples using widely available functional metagenomic methodologies^14^,^15^,^16^,^17^.

Florfenicol, a fluorinated thiamphenicol derivative, is a broad-spectrum antimicrobial agent exclusively approved for use in veterinary medicine^18^. It has been licensed in China since 1999 for the control of respiratory tract diseases and enteric infections in food-producing animals. However, the excessive use of florfenicol as an antimicrobial chemotherapeutic agent has resulted in bacterial species acquiring resistance to this agent. Since the identification of florfenicol resistance gene *floR* in the fish pathogen *Pasteurella piscicida* in 1996^19^, several specific phenicol resistance genes have been reported in florfenicol-resistant bacteria of animal origin. These genes include the phenicol-specific exporter genes *fexA*, *fexB*, and *floR*, and the multidrug resistance gene *cfr*, which encodes a 23S rRNA methyltransferase that confers resistance to phenicols as well as four other structurally unrelated antimicrobial groups (lincosamides, oxazolidinones, pleuromutilins, and streptogramin A)^20^. More recently, a novel ATP-binding cassette (ABC) transporter gene, *optrA*, which confers resistance to phenicols and oxazolidinones, was identified in *Enterococcus* and *Staphylococcus* species of both animal and human origin^21^,^22^. It is noteworthy that both *cfr* and *optrA* confer transferable resistance to linezolid. Linezolid was the first oxazolidinone to be introduced into clinical medicine to treat infections caused by vancomycin-resistant enterococci and methicillin-resistant *Staphylococcus aureus*^23^. *optrA* also confers resistance to tedizolid, which is a newly approved oxazolidinone for the management of human infections associated with Gram-positive pathogens, including linezolid-resistant strains (especially those carrying *cfr*)^24^. Although oxazolidinones have not been approved for use in the livestock or aquaculture industries, *cfr* and *optrA* are commonly detected in florfenicol-resistant bacteria of animal origin^21^,^25^. In addition to *fexA*, *fexB*, *cfr*, *optrA*, and *floR*, phenicol exporter gene *pexA* was identified in metagenomic libraries of cloned DNA isolated from Alaskan soils^14^. Interestingly, all of these genes, apart from *pexA*^14^, coexist with bacterial mobile genetic elements such as plasmids, transposons, or integrons^21^,^25^,^26^,^27^,^28^, which aid the horizontal transfer of florfenicol resistance genes (FRGs) to numerous bacterial species and genera.

Previous publications have reported the occurrence of chloramphenicol resistance genes, including *fexA*, *fexB*, *cfr*, and *floR*, in association with chloramphenicol residue in wastewater effluent from swine farm operations and corresponding wastewater-irrigated agricultural fields^29^. However, more than one decade ago, the usage of chloramphenicol in the livestock and aquaculture industries has been completely banned and florfenicol became the only available antimicrobial agent from the phenicol class in China. It is not clear whether the use of florfenicol in agriculture has contributed to the environmental accumulation of florfenicol and FRGs, especially the cfr and optrA genes also conferring resistance to other antimicrobial agents, which are critically important in the human medicine. It is very likely that the frequency of florfenicol usage in swine farms could affect the abundance and prevalence of FRGs and the accumulation of florfenicol in adjacent soils. Thus, we quantified six FRGs (*fexA*, *fexB*, *cfr*, *optrA*, *floR* and *pexA*) using quantitative polymerase chain reaction (qPCR) via a culture-independent method and detected the florfenicol residue concentrations using ultra performance liquid chromatography-tandem mass spectrometry (UPLC–MS/MS) in soils from different farms.

## Results

### Abundance of FRGs

**Figure 1:**
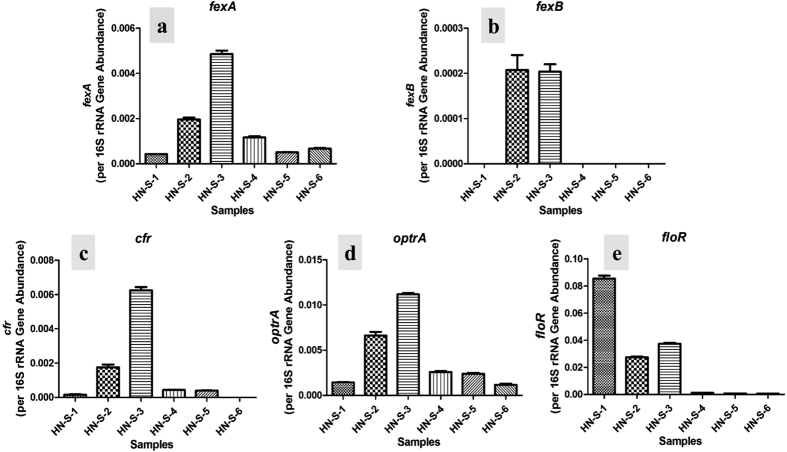
Relative abundance of five florfenicol resistance genes (a: *fexA*, b: *fexB*, c: *cfr*, d: *optrA*, and e: *floR*) in the six soil samples (target gene copies/16S rRNA gene copies). Bars represent the relative abundance of a single florfenicol resistance gene, and values shown are mean ± SE of three analytical replicates.

The florfenicol had been used to treat respiratory diseases only in farms HN-S-4, HN-S-5, and HN-S-6, while the florfenicol had been extensively used for prevention purposes in long-standing farms HN-S-1, HN-S-2, and HN-S-3. The approximate annual dosage of florfenicol used in farms HN-S-1, HN-S-2, and HN-S-3 in past three years is much more than that in farms HN-S-4, HN-S-5, and HN-S-6 (Table 1). Correspondently, our quantitative PCR result showed the relative abundance of FRGs from the farms HN-S-4, HN-S-5, and HN-S-6 was much lower than that from the farms HN-S-1, HN-S-2, and HN-S-3 (Fig. S1) and no FRGs was detected in control samples. Of the six farms, the abundance of *cfr*, *optrA*, and *fexA* was significantly higher in soils from HN-S-2 and HN-S-3 compared with the other four farms (*p* < 0.001). Interestingly, *fexB* was only detected in soils from HN-S-2 and HN-S-3 (Fig. 1), the only two farms with detectable florfenicol residue. Significantly higher levels of *floR* were detected in samples from the three long-standing farms compared with samples from the newly established farms (*p* < 0.001), with the highest abundance of *floR* detected in soils from HN-S-1 (Fig. 1). Additionally, the newly identified gene *optrA*, which confers transferable resistance to florfenicol and linezolid, was present across all soils adjacent to swine feedlots. Conversely, phenicol exporter gene *pexA* was not detected in any of the soil samples. Importantly, the relative abundance of the multidrug-resistance genes *cfr* and *optrA* was significantly higher in soils from HN-S-2 and HN-S-3 than in the other farms analyzed (*p* < 0.001).

### Metagenomic Analysis

**Figure 2:**
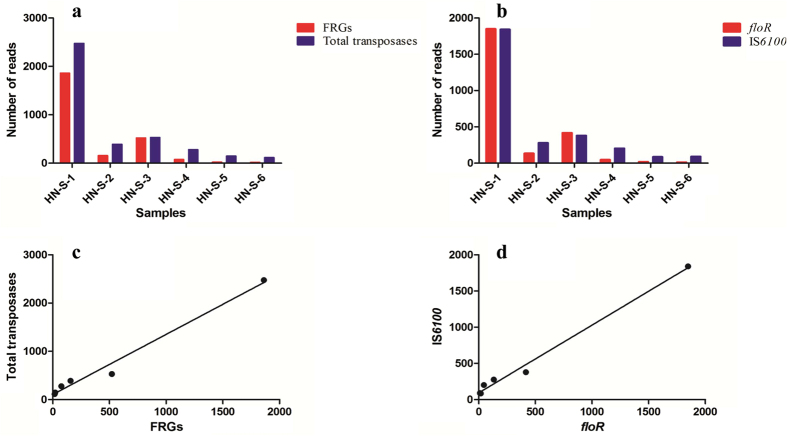
Abundance of florfenicol resistance genes (*fexA*, *fexB*, *cfr*, *optrA*, *floR*) and transposases. (**a**) Number of total reads of each of the genes in different soil samples. Red bars represent the sum of florfenicol resistance genes, and purple represents transposases. (**b**) Reads associated with *floR* and IS*6100* gene sequences in each soil sample. Red bars represent *floR* genes, and purple represents IS*6100*. (**c**) Correlation between total abundance of florfenicol resistance genes and associated transposases. (**d**) Correlation of the abundance of *floR* and IS*6100*. Sequences were analyzed using BLAT software. All figures and correlation analyses were generated using GraphPad Prism.

The absolute abundance of the six FRGs was further validated using high-throughput sequencing-based metagenomic analysis. In these six soil samples adjacent to swine farms, the number of reads identified as florfenicol resistance gene sequences was used to determine the absolute abundance. There was a significant difference in the log_2_ values for transformed FRG reads between the two different groups of farms (*p* < 0.05), which was consistent with the qPCR assays (Fig. 2a,b). Pearson correlation analyses were conducted to investigate potential relationships between the abundance of FRGs and transposases in these soil samples. Transposases, including IS*256*, IS*6100*, IS*26*, IS*1216*, IS*Enfa4*, and IS*Enfa5*, which play an important role in the horizontal transfer of FRGs between animal-associated bacteria and human clinical isolates, were identified in all soil samples. The total number of FRGs in each sample and the abundance of these genes were highly correlated with the levels of transposases in the soil samples (Fig. 2c, *r*^2^ = 0.9833, *p* < 0.0001). Furthermore, the phenicol exporter gene *floR* was highly correlated with the insertion sequence IS*6100* (Fig. 2d, *r*^2^ = 0.9912, *p* < 0.0001).

### Florfenicol Concentrations in Soil Samples

Methods were validated prior to sample analysis. To conform with the relevant validation criteria, the following parameters were used: (a) the matrix-matched calibration curves were linear in the concentration range of 0.25–20 ng g^−1^, with correlation coefficients (*r*^2^) greater than 0.999; (b) the limits of detection (LOD) and quantification (LOQ) were 0.08 ng g^−1^ and 0.25 ng g^−1^, respectively; (c) the average recoveries for florfenicol were in the range of 94.20–96.28% with relative standard deviation less than 10.5% (n = 6). Following method validation, florfenicol was detected in the soil samples from farms HN-S-2 (3.730 ± 0.327 ng g^−1^) and HN-S-3 (0.359 ± 0.028 ng g^−1^), while concentrations were below the LOQ for all other samples.

## Discussion

Our results indicated that the usage of florfenicol in the farms affect the prevalence of FRGs in the soil irrigated with the farm waste. This conclusion could be supported by the following observations: first, both the metagenomic and qPCR via culture-independent methods revealed that FRGs can be detected in the soils adjacent to the farms with florfenicol usage, but not in the control samples. Second, the prevalence and abundance of FRGs maybe affected by the amount of florfenicol used in the farms, as the farms consumed more florfenicol resulted in more detected FRGs in soils. The detectable level of florfenicol in the soils adjacent to farms HN-S-2 and HN-S-3 imply that the unabsorbed florfenicol could be excreted into the environment via manure from livestock animals.

In addition to the usage of florfenicol, many other factors could contribute to prevalence of ARGs in soils. It has been reported that the variety of ARGs and antibiotic residues in agricultural soils are correlated with soil type, manure application rate and environment conditions^30^,^31^. The long-term fertilization of antibiotic-contaminated chicken manure in greenhouse soils led to higher levels of ARGs and antibiotic residues in greenhouse soils compared with field soils^6^. The occurrence of chloramphenicol resistance genes as environmental pollution were found in manured soils^29^. Several studies also have reported that the abundance and diversity of ARGs have a marked increase in soils after repeated manure application^6^,^32^,^33^, such as multidrug resistance (MDR) genes, macrolide-lincosamide-streptogramin (MLS) genes, and sulfonamide resistance genes. Interestingly, following the use of florfenicol in these swine farms, the linezolid resistance gene *optrA* was detected in all soil samples; and the abundance of *optrA* was greater in soil samples obtained from farms HN-S-2 and HN-S-3. These were also the only two farms with florfenicol soil concentrations above the limit of quantification. However, oxazolidinones have not been approved for veterinary applications worldwide, and florfenicol is the only phenicol-derived antimicrobial agent exclusively used in selected swine farms^21^. In addition, the results imply that prior to being introduced to the farm environment, *in vivo* florfenicol can directly select for the propagation of *optrA* in bacterial isolates of animal origin. This is supported by the finding that this gene is more frequently detected in enterococci isolated from animals than from humans^21^.

Meanwhile, a rapid UPLC-MS/MS method with a lower LOD (0.08 ng g^−1^) and LOQ (0.25 ng g^−1^) values compared with previous methods^34^,^35^ was established to determine the levels of florfenicol in soil samples. To date, a number of reports have described the detection of various antibiotic residues, including tetracyclines, (fluoro)quinolones, and sulfonamides, in agricultural soils^30^,^31^. However, none of these studies described a method that is suitable for the determination of florfenicol in soil using UPLC-MS/MS. To the best of our knowledge, this is the first study to quantify florfenicol residue in soil samples collected from swine production facilities using UPLC-MS/MS. The results showed that florfenicol levels were above the LOQ on two of the long-standing farms (HN-S-2 and HN-S-3). However, the observed lower concentrations of florfenicol in soils was consistent with that published previously^35^, may be due to the adsorption, biodegradation, photolysis, infiltration of florfenicol^6^ and the dilution of florfenicol by swine manure in soils. Notably, Subbiah *et al*. confirmed that the biologically active of florfenicol could remain long-time in soils^36^ and exert a selective pressure for resistance genes in the environment. In addition, a previous study^34^ looking at the relative robustness of florfenicol showed that the half-life of florfenicol in native soil is approximately 8 days and the degradation behavior of florfenicol in the soils was connected with the activity of microbe community. Considering the rapid degradation rate of florfenicol in native soils, it is possible that the detection of florfenicol in these soil samples depend on whether the sampling sites were irrigated with florfenicol contaminated manure or not in recent days.

In general, the emergence and spread of antibiotic resistance genes are associated with mobile genetic elements, such as plasmids, integrases, and transposases^37^,^38^,^39^. To date, several insertion sequences responsible for the mobility of FRGs, including IS*6100*, IS*26*, IS*1216*, IS*256*, IS*Enfa4*, and IS*Enfa5*, have been widely detected in different Gram-positive and Gram-negative bacteria^21^,^25^,^26^,^27^,^28^. This has likely allowed the translocation of resistance genes between different plasmids, as well as mediating their integration into chromosomal DNA^25^. Importantly, in this current study, according to metagenomic analysis, the abundance of total FRGs (*fexA*, *fexB*, *cfr*, *optrA*, and *floR*) was significantly correlated with the abundance of insertion sequences (Fig. 2a,c). Of the transposases investigated as part of this study, IS*1216* is commonly located adjacent to the linezolid resistance gene *optrA* in enterococci^40^, while IS*26*, IS*1216*, IS*Enfa4*, and IS*256* have been reported to play an important role in the mobility of the multiresistance gene *cfr*^25^. In addition, the most frequently detected transposase, the IS*6100* family element, commonly coexists with complex integrons in Gram-negative bacteria, and is typically located in the flanking regions of several resistance genes, including *floR* and *tetR*^27^,^38^,^41^. We also observed a strong positive correlation between the abundance of *floR* and the presence of IS*6100* in soil samples from each of the tested farms (Fig. 2b,d). All of these results suggest that the amount of florfenicol used in the farm not only play a considerable role in the abundance and diversity of FRGs, but also simultaneously leads to the enrichment of horizontally mobile genetic elements in adjacent soils. Therefore, the effects of the application of biogas slurry and the build-up of manure generated through livestock maintenance on the microbiota of agricultural soils should not be ignored when considering the spread of antibiotic resistance genes and insertion sequences (e.g. IS*6100*). Indeed, these factors are likely to assist in the migration of FRGs through soil-dwelling bacteria and horizontally transferred mobile genetic elements, facilitating the spread to human-associated bacteria. In this study, the strong correlation between transposase enrichment and the abundance of FRGs suggests that horizontal gene transfer may have aided in the enrichment of resistance genes through the transfer of mobile genetic elements among soil bacteria.

To the best of our knowledge, this is the first study revealing the impact of florfenicol administration on the occurrence of FRGs in soil samples collected from swine farms. Our findings indicate that the amount of florfenicol used in swine husbandry could have played a considerable role in the abundance and diversity of FRGs in adjacent soils via application of swine waste. It is also the first report detailing the occurrence of the florfenicol and linezolid resistance gene *optrA* in soil samples. The results of this study suggest that soils containing *optrA*, *cfr*, and other phenicol resistance genes may act as a reservoir for florfenicol resistance. Therefore, the transfer of FRGs is also likely to affect humans that come into contact with the affected livestock, either through the food chain or through further environmental dissemination. It should be note that the selection of florfenicol and linezolid resistance genes *optrA* and *cfr* in soils, following the application of manure in swine farms with a history of administration of florfenicol, pose a significant risk to public health.

## Methods

### Sample Collection

Soil samples were collected from agricultural fields surrounding six swine feedlots in November 2014. The antibiotic use records of the six farms indicated that florfenicol had been used at each of the farms. All of the field soils were irrigated with manure every few days and had been planted with vegetable crops for human consumption. The swine farms were located in six different cities in Henan Province, China (Fig. S2) and general information regarding these pig farms is listed in Table 1. Of the six farms analyzed, HN-S-4, HN-S-5, and HN-S-6 were relatively new farms (less than 3–4 years old), while HN-S-1, HN-S-2, and HN-S-3 had been producing sows for almost two decades. Overall, the long-standing farms HN-S-1/2/3 used more florfenicol (ranging from 0.63–2.88 metric tons per year) for preventing usually once a month in the past three year than other farms HN-S-4/5/6 (ranging from 0.11–0.30 metric tons per year) for treating respiratory diseases but not prevention (Table 1). Five soil samples were collected from different locations (near the pigsty) at each farm at depths of 5–10 cm^4^ and the soils were irrigated with manure repeatedly in the past few years. Additionally, control soil sample was collected at sites >5km away from the swine farm which was manure/antibiotic free. All samples were stored in an icebox during transfer to the laboratory, and were then stored at −20 °C long term. Soil samples collected from the same farm were mixed to form a composite sample, and subsequently sieved through a 2.0-mm mesh and frozen at −80 °C for further processing.

### Quantification of FRGs

Metagenomic DNA was extracted from 0.25 g of homogenized soil using a PowerSoil DNA Isolation Kit (MO BIO Laboratories Inc., CA, USA) according to the manufacturer’s instructions, and stored at −20 °C until use. The DNA extracted from three independent replications of soil samples from each site to minimize any potential DNA extraction bias. qPCR assays were used to detect the presence of six FRGs (*fexA*, *fexB*, *cfr*, *optrA*, *floR*, and *pexA*), along with 16S rRNA, as described previously^42^. The specific primers used to amplify these gene fragments were designed using Primer 3 Plus (Table S1). Positive and negative controls were performed for each PCR reaction. The positive products were purified and ligated into vector pMD19-T (Takara, Dalian, China), and then transformed into *Escherichia coli* DH5α (Takara). DNA from recombinant plasmids containing target gene inserts was extracted using a Qiagen DNA Mini Kit (Qiagen, Hilden, Germany). The presence of the desired inserts was verified by PCR and sequencing, and then the positive plasmids were used to generate a standard curve for qPCR analysis. The qPCR assay was conducted using a QuantStudio 7 Flex Real-Time PCR System (Life Technologies, Carlsbad, CA, USA) with SYBR Premix Ex Taq II (Takara). The 20-μL reaction volume contained the following: 10 μL of SYBR Premix Ex Taq II (Til RNaseH Plus) 2*, 0.8 μL of each primer (10 nmol L^−1^), 1 μL of template DNA, and 7.4 μL of RNase-Free H_2_O (Takara). The qPCR conditions were: 95 °C for 3 min, followed by 40 cycles of 30 s at 95 °C, 30 s at 60 °C, and 30 s at 72 °C. The qPCR amplification efficiency was examined using R^2^ values (>0.999) for each calibration curve (Table S2). The amplification specificity was verified by performing a melting curve analysis (95 °C for 15 s, 60 °C for 1 min, 95 °C for 15 s) for each qPCR reaction, along with gel electrophoresis. The copy numbers of the target FRGs were quantified using a standard curve. Presence of the 16S rRNA gene was also quantified on the same plate, and the results are shown as relative abundance. To ensure reproducibility, three technique replicates for each sample were performed in parallel in each qPCR assays.

### Metagenomic Analyses

For the metagenomic analysis, six prepared soil DNA samples were sent to Berry Genomics Company (Beijing, China) for high-throughput sequencing using the HiSeq 2500 platform. The raw data were filtered following removal of low-quality reads. The filtered data were then searched against the nucleotide sequences of six florfenicol resistance genes (*fexA*, *fexB*, *cfr*, *optrA*, *floR*, and *pexA*) with a minimum 50-bp overlap length and 95% identity using BLAT software^43^. The reads that matched the florfenicol resistance gene sequences were extracted, counted, and normalized from the total reads for each sample. The reads matching major transposase genes such as IS*6100*, IS*26*, IS*256*, IS*Enfa5*, IS*Enfa4*, and IS*1216* were also extracted^25^,^27^,^28^,^38^. The flanking insert sequences were measured following metagenomic sequencing and analysis, as was florfenicol resistance gene abundance.

### Extraction Procedures and Sample Preparation

In this study, a method was developed to quantify florfenicol residues in the soil using UPLC-MS/MS. First, soil samples were thawed at room temperature. A 5-g aliquot of soil was then weighed, and 4 mL of ammonia-ethyl acetate (2 + 98, v/v) were added to extract the drug. The mixture was mixed vigorously for 2 min using a vortex and then centrifuged at 10,000 rpm for 10 min at 4 °C. The supernatant was then transferred to a 10-mL centrifuge tube containing 500 μL of an acetic acid-water solution (5 + 95, v/v) and the extraction was repeated. The supernatants were subsequently combined and evaporated at 45 °C using a gentle stream of nitrogen until the combined volume was less 500 μL. The residue was reconstituted in 2 mL of an acetic acid-water solution (5 + 95, v/v). After vortexing for 1 min, the solution was transferred to an MCX cartridge (60 mg, 3 cc, Waters, Milford, MA, USA), which was sequentially preconditioned with 3 mL of methanol and 3 mL of water. The cartridge was washed with 1 mL of the acetic acid-water solution and eluted in 3 mL of ammonia-methanol (1 + 9, v/v). The eluate was collected and evaporated completely using a gentle stream of nitrogen. The residue was subsequently reconstituted in 500 μL of acetonitrile-water (1 + 1, v/v) and centrifuged at 12,000 rpm for 15 min at 4 °C. The supernatant was filtered through a 0.22-μm nylon membrane filter and transferred into a 2-mL vial. Finally, 10 μL of the solution were injected into the UPLC-MS/MS system.

### UPLC-MS/MS Analysis

An UPLC system coupled with a Quattro LC triple quadrupole tandem mass spectrometer (Waters) equipped with electrospray ionization (ESI) was used to determine the presence, and subsequently quantify, florfenicol in the soil samples. Chromatographic separation was achieved using an Acquity UPLC BEH Shield RP18 column (50 mm × 2.1 mm, 1.7 μm) at 35 °C. Samples were separated using a mobile phase consisting of 0.1% formic acid in water (eluent A) and acetonitrile (eluent B) at a flow rate of 0.3 mL min^−1^. A linear gradient of eluent B (5–100%) was used in the total run time of 4 min (Table S3). The mass spectrometer was operated in the negative ESI mode with the following parameters: capillary voltage, 3.2 kV; cone voltage, 25 V; source temperature, 100 °C; desolvation temperature, 300 °C. Direct infusion was performed to optimize multiple reaction monitor transitions and associated acquisition parameters. The optimized conditions were as follows: m/z 356 > 336.1 (quantitative transition, collision energy, 13 eV), m/z 356 > 185.1 (qualitative transition, collision energy, 11 eV). Each sample was replicated three times for the determination of florfenicol residues.

### Statistical Analysis

The difference in the relative abundance of FRGs (target gene copies/16S rRNA gene copies) was tested in all of the soil samples using t-test (non-parametric test), with further comparison of each soil sample carried out using Unpaired t-test.

## Additional Information

**How to cite this article**: Zhao, Q. *et al*. Prevalence and Abundance of Florfenicol and Linezolid Resistance Genes in Soils Adjacent to Swine Feedlots. *Sci. Rep.* **6**, 32192; doi: 10.1038/srep32192 (2016).

## Supplementary Material

Supplementary Information

## Figures and Tables

**Table 1:** General information regarding the six pig farms sampled in this study.

Farm	Duration of operation	Breeding information			Florfenicol usage		
		Total number of animals	Annual hog output	Sows	Duration of usage	Purposes	Approx. Annual dosage in past three years (metric ton)
Farm 1/HN-S-1	19 years	158000	150000	8000	8 years	prevention	2.88
Farm 2/HN-S-2	18 years	58000	55000	3000	9 years	prevention	0.63
Farm 3/HN-S-3	20 years	37000	35000	2000	10 years	prevention	0.72
Farm 4/HN-S-4	4 years	8480	8000	480	4 years	therapy	0.11
Farm 5/HN-S-5	4 years	25600	24000	1600	4 years	therapy	0.28
Farm 6/HN-S-6	3 years	52500	50000	2500	3 years	therapy	0.30
