# Supplementary material for: Prevalence and Abundance of Florfenicol and Linezolid Resistance Genes in Soils Adjacent to Swine Feedlots
Source: Sci Rep. 2016 Aug 30;6:32192. doi: 10.1038/srep32192 (PMC5004119; doi:10.1038/srep32192)
Supplement: Supplementary Information [file srep32192-s1.pdf]

Prevalence and Abundance of Florfenicol and Linezolid Resistance Genes in Soils Adjacent to Swine Feedlots

Qin Zhao[1†], Yang Wang[1†], Shaolin Wang[1], Zheng Wang[1], Xiang-dang Du[2], Haiyang Jiang[1], Xi Xia[1], Zhangqi Shen[3], Shuangyang Ding[1], Congming Wu[1], Bingrui Zhou[4], Yongning Wu[1,5]* & Jianzhong Shen[1]*

[1] Beijing Advanced Innovation Center for Food Nutrition and Human Health, College of Veterinary Medicine, China Agricultural University, Beijing 100193, China.

[2] College of Animal Husbandry and Veterinary Medicine, Henan Agricultural University, Zhengzhou 450002, China.

[3] Department of Veterinary Microbiology and Preventive Medicine, College of Veterinary Medicine, Iowa State University, Ames, IA 50011, USA.

[4] Shanxi Key Laboratory of Ecological Animal Science and Environmental Medicine, Shanxi Agricultural University, Taigu 030801, China.

[5] The Key Laboratory of Food Safety Risk Assessment, Ministry of Health and China National Center for Food Safety Risk Assessment, Beijing 100021, China.

[†]These authors contributed equally to this work.

*For correspondences. Jianzhong Shen, Tel. (+86)-10-62732803; Fax: (+86)-10-62731032; E-mail: sjz@cau.edu.cn; Yongning Wu, Tel. (+86)-10-83235402; Fax: (+86)-10-83235402; E-mail: wuyongning@cfsa.net.cn.

Supplementary Information

SUPPLEMENTARY FIGURES

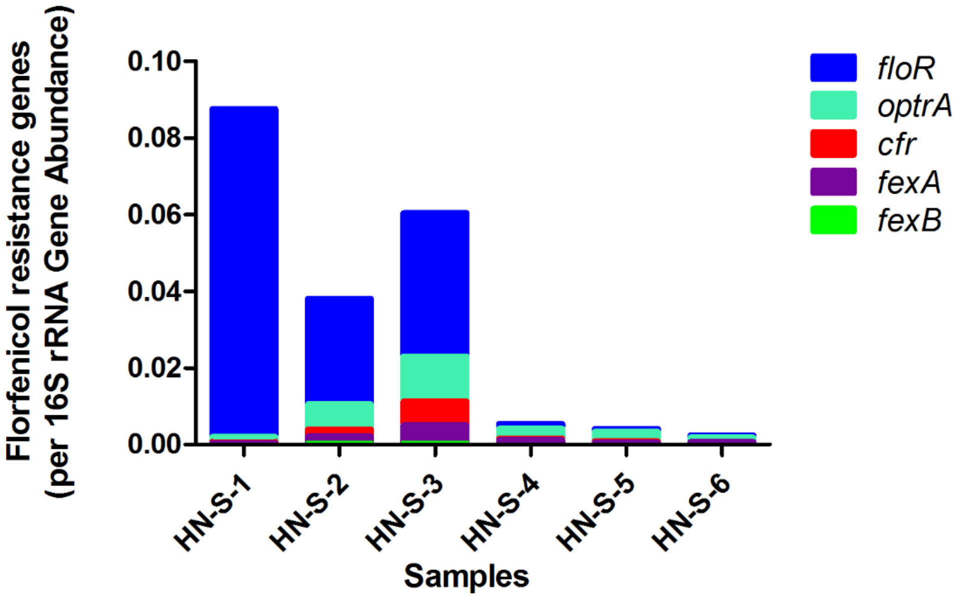

Figure S1. Relative abundance of the five florfenicol resistance genes (target gene copies/16S rRNA gene copies) in the soil samples (*pexA* was not detected in any of the samples). Bars represent the total relative abundance of the five florfenicol resistance genes (*fexA*, *fexB*, *cfr*, *optrA*, and *floR*) in soils from the different farms, with different colors indicating the relative abundance of the different genes.

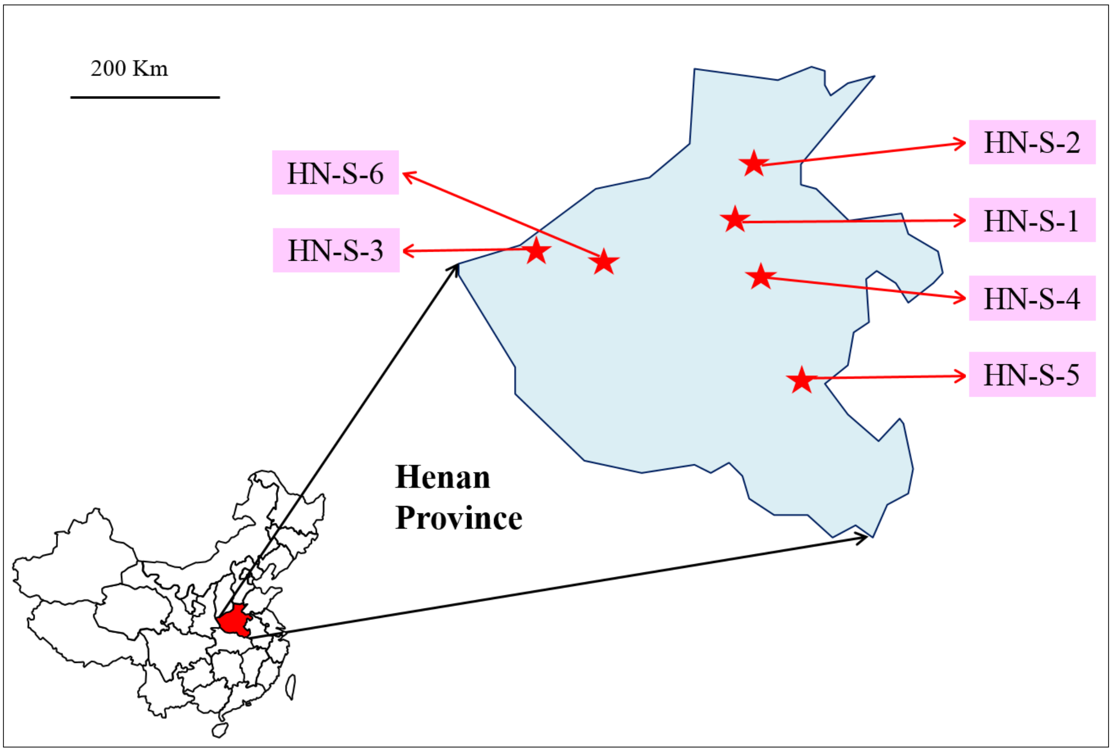

Figure S2. Locations of farms in Henan Province, China. The map of China in this figure was originated from Wikipedia under a CC-By-SA license and generated using Microsoft PowerPoint 2013 (https://creativecommons.org/licenses/by-sa/3.0/deed.en).

SUPPLEMENTARY TABLES

Table S1. Quantitative PCR primers used in this study.

Target	GenBank Accession No.	Primer (5′–3′)	Product size (bp)	References
fexA	KC989517	F:TCGCTGTTCTTGTGTTCGTC<br>R:ACAGCCCCATCAGAGTCATC	186	This study
fexB	JN201336	F:TTGGGTCGTAAGTGGTGTTG<br>R:CAGCTCCTTGAAACATTCTACC	185	This study
cfr	KC989517	F:GCAGGTTGGGAGTCATTTTG<br>R:ACGGTTGGCTAGAGCTTCAC	198	This study
optrA	KP399637	F:AAACACTTATGGGTGGTGTGG<br>R:CTGAAATGAGCCAAGAGCAG	188	This study
floR	GQ214053	F:GCTTTAGCGCCGGTATGG<br>R:GACAGTGGCGAAGGCAAAG	120	This study
pexA	HM537013	F:ACAGTGCAGGTCGAAGAACC<br>R:TGCATTACCAATCGACATCC	215	This study
16S rRNA	-	F:CGGTGAATACGTTCYCGG<br>R:GGWTACCTTGTTACGACTT	143	[1]

Table S2. Equations of standard curves and R^2.

Gene	Equations	R^2
fexA	y = -0.2599x + 11.056	>0.999
fexB	y = -0.2654x + 10.879	>0.999
cfr	y = -0.2739x + 10.918	>0.999
optrA	y = -0.2532x + 11.182	>0.999
floR	y = -0.3011x + 11.524	>0.999
16S rRNA	y = -0.2630x + 11.181	>0.999

Table S3. Liquid chromatography gradient program (mobile phase).

Time	Flow (mL/min)	Solvent A (%)	Solvent B (%)	Curve
0.00	0.3	95	5	-
0.50	0.3	95	5	6
1.00	0.3	30	70	6
2.00	0.3	30	70	6
2.10	0.3	0	100	6
2.50	0.3	0	100	6
4.00	0.3	95	5	1

Solvent A: water with 0.1% formic acid; Solvent B: acetonitrile.

References

1. Gaze, W.H. et al. Impacts of anthropogenic activity on the ecology of class 1 integrons and integron-associated genes in the environment. *ISME J* **5**, 1253-61 (2011).
